# Discontinuation of long-term care among persons affected by the 2018 Japan Floods: a longitudinal study using the Long-term Care Insurance Comprehensive Database

**DOI:** 10.1186/s12877-022-02864-4

**Published:** 2022-03-01

**Authors:** Daisuke Miyamori, Shuhei Yoshida, Saori Kashima, Soichi Koike, Shinya Ishii, Masatoshi Matsumoto

**Affiliations:** 1 Department of General Internal Medicine, Hiroshima University Hospital, Hiroshima, Japan; 2 Department of Community-Based Medical System, Graduate School of Biomedical and Health Sciences, Hiroshima University, Hiroshima, Japan; 3 Environmental Health Sciences Laboratory, Graduate School of Advanced Science and Engineering, Hiroshima University, 1-5-1 Kagamiyama, Higashi-Hiroshima, Hiroshima, Japan; 4 Division of Health Policy and Management, Center for Community Medicine, Jichi Medical University, 3311-1 Yakushiji, Shimotsuke, Tochigi Japan; 5 Department of Medicine for Integrated Approach to Social Inclusion, Graduate School of Biomedical and Health Sciences, Hiroshima University, Hiroshima, Japan

**Keywords:** Long-term care discontinuation, Disaster, Database claims, Facility closure, Rural health services

## Abstract

**Background:**

Most older people with disabilities or illnesses continue to use long-term care (LTC) services for the rest of their lives. However, disasters can cause a discontinuation of LTC services, which usually means tragic outcomes of affected persons. In view of the recent progression of population aging and the increase in natural disasters, this study focuses on the impact of disasters on older people’s discontinuation of LTC services, and those more risk of such discontinuation than others. However, current evidence is scarce.

**Methods:**

We conducted a retrospective cohort study with 259,081 subjects, 2,762 of whom had been affected by disaster and 256,319 who had not been affected during the 2018 Japan Floods. The sample in the three most disaster-affected prefectures was drawn from the Long-term Care Insurance Comprehensive Database and included older people certified with care-need level. The observation period was two months before the disaster and five months after it. We calculated the hazard ratio (HR) of municipality-certified subjects affected by the disaster versus those who were not. Subgroup analyses were conducted for categories of individual-, facility- and region-associated factors.

**Results:**

Affected persons were twice as likely to discontinue LTC services than those who were not affected (adjusted HR, 2.06 95% CI, 1.91–2.23). 34% of affected persons whose facilities were closed discontinued their LTC services at five months after the disaster. A subgroup analysis showed that the risk of discontinuing LTC services for affected persons compared to those who were not affected in the relatively younger subgroup (age < 80: adjusted HR, 2.55; 95% CI, 2.20–2.96 vs. age ≥ 80 : 1.91; 1.75–2.10), and the subgroup requiring a lower level of care (low: 3.16; 2.74–3.66 vs. high: 1.71; 1.50–1.96) were more likely to discontinue than the older and higher care level subgroups.

**Conclusions:**

A natural disaster has a significant effect on the older people’s discontinuation of LTC services. The discontinuations are supposedly caused by affected persons’ death, hospitalization, forced relocation of individuals, or the service provider’s incapacity. Accordingly, it is important to recognize the risk of disasters and take measures to avoid discontinuation to protect older persons’ quality of life.

**Supplementary Information:**

The online version contains supplementary material available at 10.1186/s12877-022-02864-4.

## Introduction

Building a mature welfare state is an urgent task for many countries because of the rapid progression of aging in societies. People under welfare services are quite vulnerable to disasters, and in the context of the increasing trend of large-scale natural disasters at a global level, every mature welfare state should build a welfare system that is robust to disaster impacts [1]. Japan has one of the world’s highest proportion of older people in its population and also one of the highest frequencies of natural disasters [2]. Over five million older people in Japan are currently using care services based on the long-term care (LTC) insurance system, which is a public, nationwide comprehensive welfare insurance scheme covering most of the care services of the entire older population [2]. Every year various types of disasters, such as typhoons, earthquakes, and floods, hit Japan, and their frequency and scale are increasing due to global climate change. Older people, particularly those who are using care services, are vulnerable to disasters because such disasters affect their physical and mental condition, support from family members, and service provisions by professional care providers.

### Catastrophic rainfall in western Japan

From the end of June to the beginning of July 2018, a series of record-breaking deluges devastated a wide range of area in western Japan [3]. Among the most severely affected areas were Hiroshima, Okayama, and the Ehime prefecture. About 89.4% of all fatalities and 76.8% of all injuries occurred in the above three prefectures. Parts of these prefectures received more than 360 millimeters of precipitation in only two hours, and a total rainfall of more than 500 millimeters. During the disaster, floods and landslides caused severe damage to the local infrastructure and resulted in electricity shortages and a lack of water at 75,000 and 260,000 houses, respectively, for one month after the deluge. Additionally, more than 4,000 houses in these three prefectures were inundated, and complete and partial damage occurred in approximately 6,700 and 11,000 houses, respectively. Two million people were evacuated, 237 died, eight were missing, and 433 were injured [3]. A number of nursing care services, including home visit services, day care, short-stay services, and in-facility services, were affected and were unable to continue providing services. The total financial damage was estimated at approximately 1.3 billion yen (USD 1.2 million) [3]. This was the second-largest natural disaster in the past 100 years in Japan, exceeded only by the Great Eastern Japan Earthquake. The Cabinet Office designated this as an “extremely damaging disaster” under the Law Concerning Special Fiscal Aid for Coping with Disasters.

### LTC insurance claims in Japan

The LTC insurance system covers all formal and fee-charging care services, and provides support according to the degree of disability, with the aim of enabling independent living [4, 5]. It is a social insurance system governed by each municipality wherein all residents aged 40 or older must pay a monthly premium. Residents older than 40 with a disability from specific diseases and residents older than 65 years with a disability from any disease are eligible to receive the services [6]. Out-of-pocket charges for LTC services vary from 10 to 30% of the total charge, depending on the recipient’s income level, but it never exceeds 44,000 yen (USD 420) per month. Users are classified into seven grades according to the severity of their disabilities. The amount of care services they may receive varies depending on the level of care needed. The seven grades include support levels 1 and 2, defined as those who maintain the basic activities of daily living (ADL) but need some care, and certified care-need levels 1–5, defined as those who have lost the basic ADL and need substantial care. The higher the level, the more services the person can use. The three major reasons for care-need level certification are dementia (24.8%), stroke sequelae (18.4%), and frailty (12.1%) [7].

### Disaster and discontinuation of LTC services

High mortality and morbidity rates have been reported among older people and people with high care needs in areas affected by world-class disasters, such as the Great East Japan Earthquake and Hurricane Katrina [8–12]. Death and severe illness among the victims of such disasters can lead to a temporal or terminal ending of LTC service use. These disasters can damage care facilities in the affected areas, which results in a stoppage of service utilization. Therefore, during such disasters, those affected are probably more likely to be forced to stop using LTC services than non-affected persons. Normally, once an older individual starts using LTC services, he or she usually continues to use such services until the end of his or her life. Thus, a discontinuation of service use means something catastrophic has happened. The major reasons for discontinuation of LTC services are: (1) death, (2) hospitalization of the user, (3) incapacity of the service provider due to disaster damage, and (4) forced relocation. All of these outcomes mean a significant loss and burden for an older person and/or their family, and should therefore be avoided whenever possible.

To the best of our knowledge, however, no studies have investigated whether or not a disaster actually caused the discontinuation of LTC services. Furthermore, little is known about which individual, community, or institutional characteristics amplify the effect of a disaster on the discontinuation of LTC. The lack of evidence is probably because the government’s database on LTCI has only recently been available for research purposes, and only a few major disasters have occurred since that time.

Some studies, both within and outside Japan, have investigated the impact of natural disasters on a specific aspect of the misfortune care recipients have experienced, such as hospitalization, emergency room visit, and death [13, 14]. However, most of these studies have examined the impact by comparing the affected areas or affected facilities with non-affected ones without examining the impact on each individual. These community- and facility-based ecological studies are prone to a failure called “ecological fallacy,” in which biased results can be obtained by making inferences about an affected individual based on aggregate data. In order to overcome the limitation of these studies, an individual-level cohort study is needed that identifies each affected and non-affected individual within a disaster area to make a direct comparison between them. Other limitations of previous studies are a small sample size, a large amount of missing data, a short observation period, and incomplete demarcation of the study areas [15]. Thus, comprehensive data is needed that covers an entire impacted area and all the individuals affected over a sufficient length of time to establish a more precise understanding of the effects of a disaster.

The purpose of this study was to examine whether severely affected persons were more likely to discontinue LTC services than less severely affected persons by identifying those in the database who suffered significant housing and economic damage. For convenience sake, the term “affected persons” refers to those who were certified as affected by the disaster, while “non-affected persons” refers to residents of the study area who were not certified as affected by the disaster.

In this cohort study, we used the Long Term Care Insurance Comprehensive Database provided by the government, in which affected individuals and non-affected persons of the 2018 Japan Floods are identifiable. Based on the data, the frequencies of discontinuation of LTC services among those affected and non-affected in the three hardest-hit prefectures were calculated and compared with each other. In addition, individual, facility, and regional factors that magnified the effect of the disaster were identified. Based on these results, we discuss political options for Japan and other welfare states to prevent disruption in the administration of care caused by large-scale disasters.

## Materials and methods

### Study design and settings

This is a retrospective cohort study that covers Ehime, Hiroshima, and Okayama prefectures, which in June 2018 had a total population of approximately six million (4.7% of the national population) [16]. The observation period was from May 1, 2018, through December 31, 2018, which was the period between two months before and six months after the disaster.

The inhabitants included a substantial number of the older population; 30.1% (n = 1,829,000) aged > 65, and 9.8% (n = 593,000) aged > 80 years. These proportions were similar to those among the national population (28.1% and 8.7%, respectively) [16].

### Eligibility criteria

Residents living in these three prefectures, who had a certified level of care need as of May 2018, and who were using any LTC service during the study period, were included in this cohort. We excluded residents who did not use any service throughout the study period and those who discontinued before the disaster.

Data were derived from the Long-Term Care Insurance Comprehensive Database, which is maintained by the Japanese Ministry of Health, Labour, and Welfare (MHLW). For this research, we extracted all the data from the database for the above mentioned period with special permission from the MHLW (approval no. 0711-1).

### Outcome

The primary endpoint was the discontinuation of LTC services, including all home visit services, day services, short-stay services, and in-facility services (residential services in nursing homes). LTC discontinuation was recognized when those in the sample stopped using these LTC services for more than two months. In this study, the reasons for discontinuation were not recorded, except for residents using in-facility services. The considerable main reasons for discontinuing LTC services were death, admission to hospital, incapacity of service providers, change in resident registration to another address, and recovery from a dependent to an independent status, although the last item is quite unlikely in light of the usual aging process.

In this study, the personal identification number on the database was changed when a person moved from one municipality to another and changed their resident registration, whereas the identification wasn’t changed when a person moved but did not change their registration. Therefore, only when residents had changed their resident registration and could not be tracked in the database were they considered as having discontinued LTC services.

### Certification of severely affected persons

In this study, persons severely affected by the disaster were certified as such by the local government [17]. After the 2018 Japan Floods, the government enacted the *Disaster Relief Act*, and certified those affected as fully exempt from out-of-pocket payment of charges for any LTC service. A certified affected person needed to meet at least one of the following conditions: (1) the person’s house was damaged completely or partially; and (2) the main caregiver, who financially supported the person, was severely injured, lost his/her job, passed away, or was missing due to the disaster. The certification was conducted by each municipality. Severely affected persons with disaster certification were identified in the database if they were fully exempted from out-of-pocket payment of charges.

As this disaster was designated “extremely damaging,” the MHLW instructed nursing homes to certify residents as severely affected only by verbal confirmation if they met the conditions for disaster certification, and exempted them from applying to be certified as severely affected until January 2019.

### Closure of care facility

A temporary or permanent closure of a service facility can shorten the life expectancy of a disaster-affected person [18, 19]. If an LTC facility stops providing services, residents who use the facility are forced to relocate to another service facility or resort to family care, which substantially affects a person’s health and life thereafter. In this study, therefore, we assessed the risk of those who experienced closure of their residential facility on discontinuing LTC. In the database, a facility was recognized as closed if it stopped requesting any payment claim from the LTC insurer for more than two months.

### Potential confounding factors

Information on individual, facility, and regional factors were also collected from the database. Individual factors were subjects’ age, gender, and level of care needed. Age was divided into eight categories: less than 65 years old, every five years from 65 years to 95 years old, and above 95 years old. Level of care need was determined by the local government where the subject lived, based on opinions issued by a family physician and care experts, based on objective data regarding the subject’s physical, psychological, and social conditions. The certified level is known to be well correlated with the activities of daily living and level of cognitive impairment [20–22].

A facility-associated factor was the type of services LTC facilities provided. The type of service was categorized based on the service code in the database (Supplementary Table 1).

The municipality (city, town, village) code for the area in which each subject lived was extracted from National Census Data. Municipalities in the study area were divided into two categories: cities and towns/villages [23]. Regional factors such as: population density, number of nursing homes per 10,000 population, hospital beds per 10,000 population, average income, and proportion of older people among the whole population, were obtained from National Census Data [23].

### Statistical methods

Data on the baseline characteristics in both disaster-affected and non-affected persons were presented as the mean and standard deviation for each continuous variable and as the number and proportion for each categorical variable. A chi-square or Fisher’s exact test was used to detect the difference in a dichotomous variable between the two groups. Student’s t test or the Wilcoxon rank-sum test was applied to compare affected and non-affected persons in terms of each continuous and categorical variable.

The event-free rate, or proportion of those who did not experience an interruption in LTC service use, was estimated with the Kaplan-Meier survival analysis, and survival estimates were compared between affected and non-affected persons using the log-rank test. Data were censored on December 31, 2018. Patients who were lost were censored at the month of last follow-up. The event-free survival rate was calculated from July 1, 2018 to the month of the discontinuation of LTC services. Because the database has monthly-aggregated data of payment claims and service usage, we could not calculate the exact days from the disaster to when discontinuation took place.

The hazard ratio (HR) of affected versus non-affected persons who experienced LTC discontinuation was calculated using a Cox proportional hazard model. HRs were calculated with three models: a crude model, an age and sex-adjusted model, and a model adjusted for factors related to the individual, facility, or region (multivariate-adjusted model). The findings of the above three models were compared between a complete case analysis and multiple imputation analysis with a chained equation [24].

Reasons for discontinuation of LTC services were available only among the subjects who used in-facility services before the discontinuation. The coded reasons were “death”, “hospitalization”, “transfer to another facility”, “return to home”, and “others”. We compared the distribution of the reasons between victims and non-victims, using chi-square test for overall difference and residual analysis for the contributions of each reason [25].

Furthermore, we calculated the interaction term between affected status and each subgroup in the multivariate-adjusted model to conduct the likelihood ratio test wherein the term was compared with each main result of the multivariable Cox proportional hazards model. In the same subgroup, a five-month, cumulative event-free rate and its 95% confidence interval were determined.

Data were analyzed using Stata version 16.1 (StataCorp LP, College Station, TX). A *P*-value < 0.05 (two-tailed) was considered statistically significant.

### Ethical considerations

 The study was conducted according to the guidelines of the Declaration of Helsinki, and the study protocol was submitted and approved by the Ethical Committee for Epidemiology of Hiroshima University (approved no. E-1389). The need to obtain informed consent from each subject was waived, as this was a retrospective review of the subjects’ records according to the Ethical Guidelines for Medical and Health Research Involving Human Subjects in Japan.

## Results

### Subjects

**Fig. 1:**
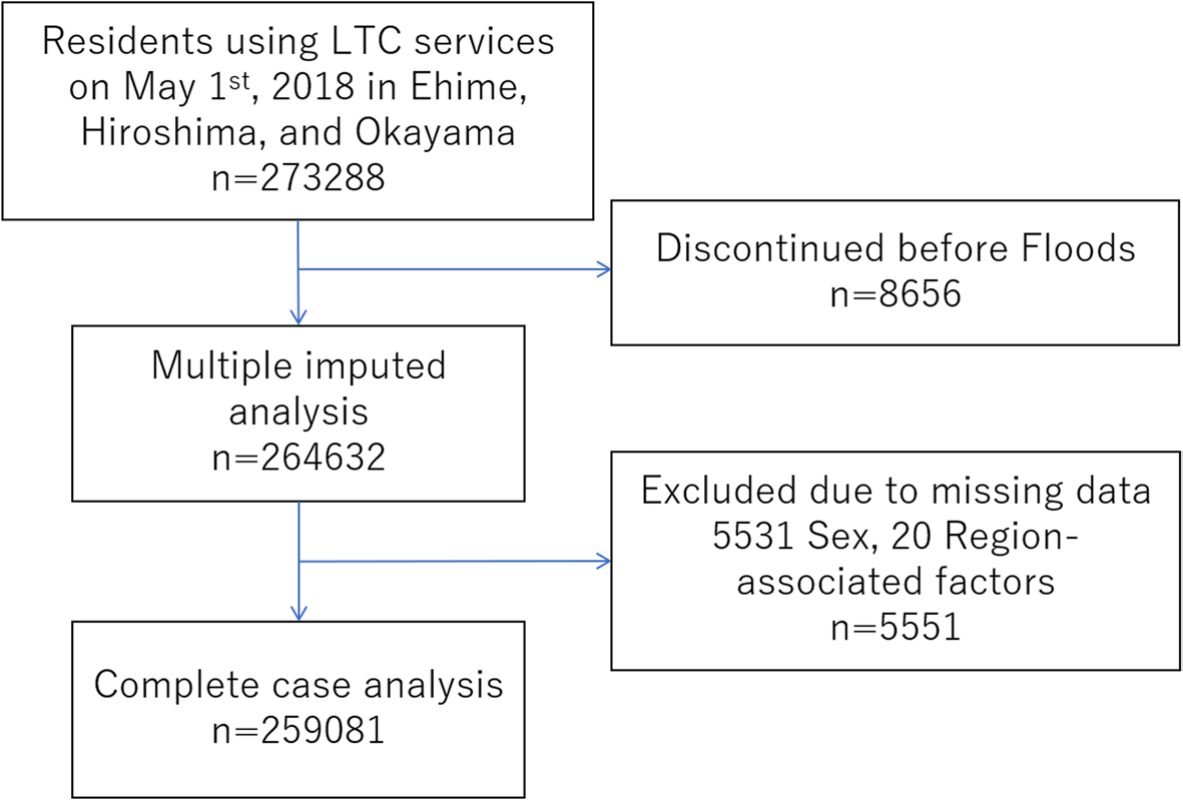
Flow chart for the selection of participants. On May 1st, 2018, 273,288 subjects living in three prefectures were included in this study. 8,656 subjects discontinued long-term care before the 2018 Japan Floods and 5,551 subjects were excluded from the primary analysis due to missing data

Figure 1 shows the recruitment process of the study subjects. In May 2018, 273,288 people used LTC services. Of these, 8,656 were excluded because they had discontinued LTC services by the day of the disaster (July 1, 2018). Among the 264,632 cases remaining, 5,551 were excluded due to a lack of data in at least one variable. Finally, 259,081 people were included as qualified subjects for the complete case analysis.

Table 1 shows the baseline characteristics of the study subjects. The median age category of the subjects was 85–90 years old, and men accounted for 28.4% of all participants. Among these subjects, 1.1% were confirmed as disaster-affected persons. Closure of a facility in use was observed in 3,857 (1.5%) of the subjects. Age category and level of care needs were significantly lower in disaster-affected persons. Affected persons used in-facility care, multifunctional facility, and home visit services more frequently than non-affected persons. Regional factors, such as hospital beds per population, average income, number of nursing homes and the proportion of older people were significantly higher in disaster-affected than non-affected persons.

**Table 1:** Baseline Characteristics of Study Participants

	Total (*N* = 259,081)	Disaster affected (*n* = 2,762, 1.1%)	Non-Affected (*n* = 256,319, 98.9%)	*P* value
Individual-associated factors				
Age, categorical (%)				<0.001†
<65	4,493 (1.7)	58 (2.1)	4,435 (1.7)	
65-69	9,403 (3.6)	99 (3.6)	9,304 (3.6)	
70-74	15,987 (6.2)	187 (6.8)	15,797 (6.2)	
75-79	28,407 (11.0)	333 (12.1)	28,074 (11.0)	
80-84	53,672 (20.7)	576 (20.9)	53,096 (20.7)	
85-90	72,126 (27.8)	793 (28.7)	71,333 (27.8)	
90-94	52,476 (20.3)	510 (18.5)	51,966 (20.3)	
>95	22,520 (8.7)	206 (7.5)	22,314 (8.7)	
Men, n (%)	73,555 (28.4)	842 (30.5)	72,713 (28.4)	0.01^*^
Level of care needs (%)				<0.001†
Support level 1	20,456 (7.9)	265 (9.6)	20,191 (7.9)	
Support level 2	27,030 (10.4)	370 (13.4)	26,660 (10.4)	
Care need level 1	58,622 (22.6)	659 (23.9)	57,963 (22.6)	
Care need level 2	51,328 (19.8)	532 (19.3)	50,796 (19.8)	
Care need level 3	39,788 (15.4)	360 (13.0)	39,428 (15.4)	
Care need level 4	34,406 (13.3)	316 (11.4)	34,090 (13.3)	
Care need level 5	27,451 (10.6)	260 (9.4)	27,191 (10.6)	
Facility-associated factors (%)				
Closure of facility in use	3,857 (1.5)	416 (15.1)	3,441 (1.3)	<0.001^*^
Type of LTC services in use				
Home visit service	81,443 (31.4)	797 (28.8)	80,646 (31.5)	0.005^*^
Day care	139,073 (53.7)	1,527 (55.3)	137,546 (53.7)	0.055^*^
Short term stay	70,386 (27.2)	917 (33.2)	69,469 (27.1)	<0.001^*^
Nursing facilities	104,332 (40.3)	1,035 (37.5)	103,297 (40.3)	0.004^*^
Multifunctional facility	9,124 (3.5)	71 (2.6)	9,053 (3.5)	0.006^*^
Care manager service	39,991 (15.4)	290 (10.5)	39,701 (15.5)	<0.001^*^
Region-associated factors				
Population density (SD)	2,017 (1,901)	1,510 (1,399)	2,023 (1,905)	<0.001‡
Hospital beds per population (SD)	1.5 (0.6)	1.6 (0.7)	1.5 (0.6)	0.009‡
No. of nursing facilities per 10,000 elderly	23 (1.1)	25 (1.1)	23 (1.1)	<0.001‡
Average income (every 1,000 yen) (SD)	2,971 (276)	2,916 (277)	2,972 (276)	<0.001‡
Proportion of the elderly (%)				<0.001†
Low 19.8-25.0	97,601 (37.7)	599 (21.7)	97,002 (37.8)	
Middle 25.5-30.7	77,937 (30.1)	1,102 (39.9)	76,835 (30.0)	
High 31.2-49.1	85,543 (33.0)	1,061 (38.4)	83,543 (33.4)	

Individual- and facility-associated factors were extracted from the Long-term care insurance comprehensive database, and region-associated factors were extracted from the National Census Database. *LTC* long-term care, *SD* standard deviation, *No.*, number; ^*^ chi-squared test, † Wilcoxon rank-sum test, ‡ two samples t-test

### Association between the disaster and health outcomes

**Fig. 2:**
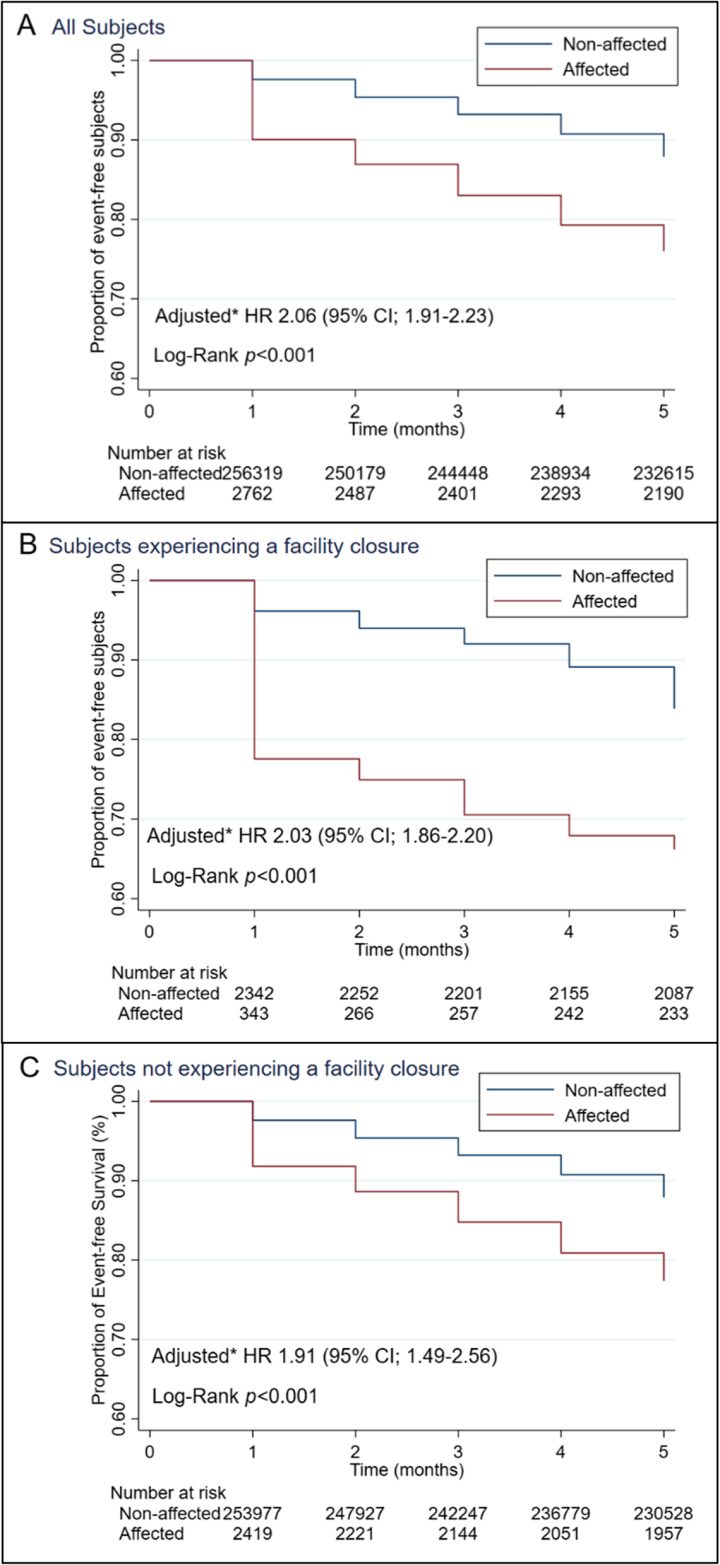
Kaplan-Meier Curves for Event-free Survival and Hazard Ratios of Disaster Affected Persons for LTC Discontinuation. Data show event-free rate among disaster affected persons compared with non-affected in all subjects (Panel A), in subjects with facility closure (Panel B) and in subjects without facility closure (Panel C). No interaction was found between disaster-affected persons and closure of associated service facility (p = 0.16). CI, confidence interval; HR, Hazard ratio; *Adjusted for age, sex, level of care needs, type of LTC services in use, facility closure, population density, average income, older people rate of the living place

Figure 2 shows the cumulative event-free rate of LTC discontinuation among affected and non-affected persons. The figures also show the HRs of affected persons for discontinuation. Among all subjects, 663 affected and 31,092 non-affected persons discontinued their use of LTC services after the disaster, which means that affected persons were twice as likely to discontinue LTC services than non-affected persons (adjusted HR, 2.06; 95% CI, 1.91–2.23) after adjusting for factors related to the individual, facility, and region. When the analysis was stratified according to the existence/non-existence of closure of a facility, the risk of affected persons discontinuing LTC was approximately double that of non-affected persons (adjusted HR 2.03 for those without facility closure and 1.91 for those with facility closure). In the group with a facility closure, the majority who discontinued LTC did so immediately after the disaster, while in the group without a facility closure the outcome took place throughout the study period. The result was robust throughout the three different models we conducted: the crude model (HR, 2.15; 95% CI, 2.00-2.33), the age and sex-adjusted model (HR, 2.15; 95% CI, 1.99–2.32), and the multiple imputed model (adjusted HR, 2.06; 95% CI, 1.91–2.23) (Supplementary Table 2). In the multivariable adjusted model, older age and higher level of care needs were significantly associated with discontinuation of LTC services, even after being adjusted for disaster status.

Table 2 shows the incidence rate, incidence rate ratio, and event-free rate sorted by a combination of disaster status and facility closure status. The incidence rates per 1,000 person-months among persons severely affected by the disaster who experienced facility closure and non-affected persons who did not experience facility closure were 86.5 and 25.4, respectively, with the incidence rate ratio of those who were severely affected by the disaster and experienced facility closure being 3.41 (95% CI; 2.82–4.09). Approximately one-third of the group that were severely affected by the disaster and experienced facility closure discontinued LTC services during the five months after the floods.

**Table 2:** Incidence Rate and 5-month Event-free Survival Rate of LTC Discontinuation

	Incidence rate^a^ (95% CI)	IRR (95% CI)	EFR in 5 mo (95% CI)
Disaster affected w/ FSC (*N* = 416)	86.5 (72.1-103.8)	3.41 (2.82-4.09)	66.2% (60.9-70.9)
Disaster affected w/o FSC (*N* = 2762)	50.7 (46.6-55.1)	2.00 (1.83-2.18)	77.4% (75.7-79.0)
Non-affected w FSC (*N* = 3441)	34.2 (31.0-37.9)	1.35 (1.22-1.50)	83.9% (82.3-85.3)
Non-affected w/o FSC (*N* = 256319)	25.4 (25.1-25.6)	Ref	87.9% (87.8-88.0)

^a^Per 1,000 person months; *CI* confidence interval, *LTC* long-term care, *IRR* incidence rate ratio, *mo,* months, *FSC* facility closure, *EFR* event-free rate, *w/* with, *w/o* without

Table 3 shows reasons for discontinuing LTC services for residents using in-facility services. regulations for the use of the database prohibit the use of numbers less than 10, which may identify individuals. Therefore, only percentages are given for Affected person and Non-affected person. The chi-square test for reasons of discontinuation were significantly different between affected and non-affected persons (*p* < 0.001). However, the proportion of subjects who were discharged from the facility to home and subsequently used no service (the group considered to have fully recovered) was only 2.1% among non-affected and 2.2% among people affected by the flood; the difference was not significant in residual analysis (*p* = 0.40). The proportion of death cases was significantly higher among non-affected than affected people (*p* < 0.001), while the proportion of hospitalization was not difference between the two groups (*p* = 0.29).

**Table 3:** Reasons for Discontinuation of LTC Services in the Affected and non-Affected Persons using in-Facility Services

	Total (*N* = 9131)	Affected persons ^a^	Non-affected persons ^a^	*p*-value for Chi-square test	*p*-value for Residual analysis
The reason of discontinuation (%)				<0.001	
Death	4958 (54.3 %)	25.9 %	54.7 %		<0.001
Admission to hospital	3357 (36.8 %)	40.0 %	36.7 %		0.29
Moved to other facilities	88 (1.0 %)	8.1 %	1.1 %		<0.001
Becoming fully independent	195 (2.1 %)	2.2 %	2.1 %		0.40
Others	522 (5.7 %)	23.7 %	5.4 %		<0.001

*LTC* long-term care; ^**a**^ Regulations for the use of the database prohibit the use of numbers less than 10, which may identify individuals. Therefore, only percentages are given for Affected person and Non-affected person

### Subgroup Analysis

**Fig. 3:**
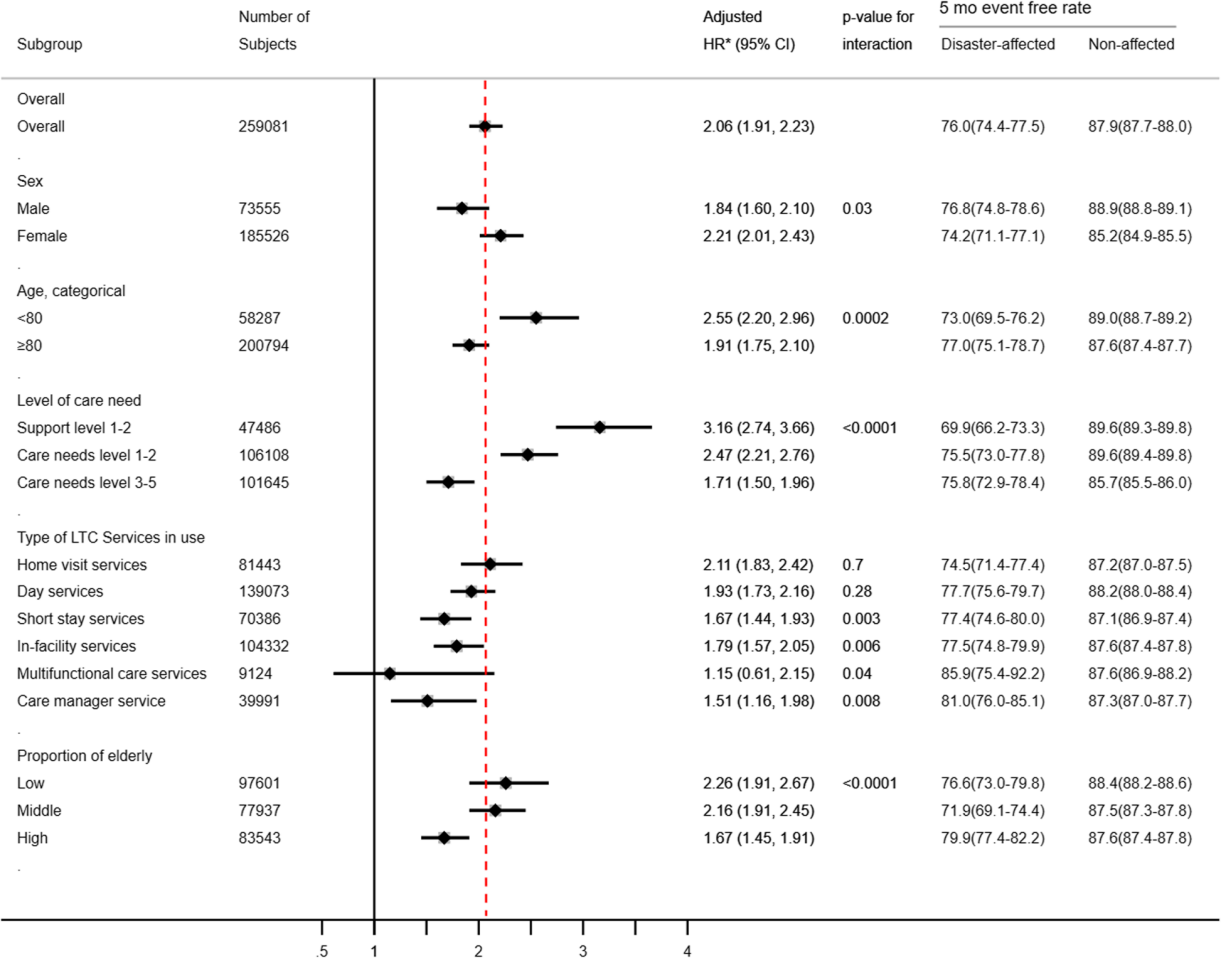
Hazard ratio of affected persons for LTC discontinuation and 5-month event free rate in each subgroup of subjects. Each hazard ratio was adjusted for all the other variables. Test for interaction between disaster suffering and each subgroup was conducted. HR, hazard ratio; CI, confidence interval; mo, months

Subgroup analysis showed that the risk of persons affected by the disaster discontinuing LTC services was significantly higher than non-affected persons in most of the subgroups (Fig. 3). In particular, hazard ratios were higher in the categories aged less than 80 and care support 1–2, compared with those aged 80 or older and those who had a care needs level of between 3 and 5, respectively. The risk of users of a multifunctional care service was not significantly different from non-users (adjusted HR 1.15; 95% CI, 0.61–2.15). There were significant interactions between disaster status and age category, level of care needs, sex, and specific LTC services, such as short-stay services, in-facility services, and multifunctional care services.

## Discussion

After controlling for factors related to the individual, facility, and region, this retrospective cohort study revealed that persons severely affected by the 2018 Japan Floods were twice as likely to discontinue LTC than non-affected persons were. This result was robust in multiple imputation analysis. Moreover, the rate of discontinuing LTC among disaster-affected persons who had also experienced a facility closure was strikingly high (86.5 events per 1,000 person-months). One quarter of those affected by the disaster discontinued LTC services over the five-month period after the disaster; and this value increased to one third among those that were affected by the disaster and experienced closure of their care facilities. To the best of our knowledge, this is the first large-scale longitudinal study showing the effect of a natural disaster on the continued use of care services.

Discontinuation of LTC is usually due to death, hospitalization, or forced relocation on the part of the care recipient, and closure of the service facility on the part of the provider. In Hurricane Katrina, the incidence of death at 30 and 90 days after the disaster among nursing home residents was higher than that of previous years [14]. This increase in disaster-related mortality may partially explain the post-disaster increase in LTC discontinuation shown in our study. Another potential reason for LTC discontinuation is hospitalization. Our findings are supported by previous studies showing that hospitalization rates increased in the entire affected area during Hurricane Katrina [13, 14, 26]. Forced relocation and facility closure entailed separating older people from their familiar environment, which placed a heavy physical and psychological burden on them due to the destruction of the living environment, and the loss of caregivers [27, 28]. A previous facility-based study reported that the risk of death and hospitalization increased among facility residents who were forced to relocate due to Hurricane Katrina and the Great East Japan Earthquake [18, 19, 29]. However, these previous reports were based on studies with a cross-sectional or ecological design, which have difficulty in confirming the association between the disaster and its individual-level effect. Our study overcame the limitation of past studies and strongly suggests that a natural disaster has a significant impact on the quality of life of those affected by the disaster.

In order to reduce the impact of disasters on care recipients, local governments have been conducting disaster preparedness campaigns such as encouraging recipients to evacuate early, educating them about evacuation sites, and assisting the development of community support programs. For a further reduction of facility-derived care discontinuation, local governments should increase the capacity of residential care facilities and strengthen the supply of LTC services. In Japan, however, the capacity of residential care facilities has not been able to keep pace with the rapid increase in the number of people in need. There is also a serious shortage of human resources to provide long-term care [22, 30], which has forced Japan to utilize care workers from overseas to address the demand-supply gap [31]. Prompt improvement of this situation is essential for Japan to develop into a disaster-resilient welfare nation.

Meanwhile, in the subgroup analysis, the risk of LTC discontinuation was higher among those aged less than 80 and support level 1 and 2, compared to the other subjects, and this result differs from the general assumption that older age and ADL decline are the risks of LTC discontinuation. Several factors can explain this gap; (1) care interruption caused by destruction of homes and loss of family caregivers, (2) increased hospitalization due to worsened cognitive function, physical function, frailty, and other health conditions after the disaster [14, 32, 33], (3) loss of care services due to disrupted infrastructure [34, 35], and 4) reallocation of care resources to people with a high level of care needs. Older people who do not live in nursing homes are dependent on the community-based services such as home care and care by family caregivers. In contrast to the in-facility people who are fully supported by facility’s caregivers, those at home tend to be cut off from the community care network and be isolated after a disaster. Also the disaster itself can cause cognitive decline, physical impairment, and the progression of frailty. In particular cognitive function of people receiving home care is reportedly more vulnerable to disasters than that of residents in LTC facilities [36], which supports the gap observed in this study. Furthermore, the disruption of infrastructure during the disaster can lead to a decreased supply of LTC services. Previous studies have shown that disaster sufferers need more LTC services than usual for several months after the disaster [37], and the expanded gap between supply and demand in LTC services must be met through shifting services to those with higher level of care needs. As a result, those with low levels of care needs and relatively younger older people might have led to LTC discontinuation more often than the older elderly and those with high-level of care needs. Therefore, it is important to pay due attention not only to very old people and the population that has high care needs, but also to relatively younger people and those who are on the middle and low levels of care needs, who have conventionally been regarded as a less vulnerable group of people during a disaster.

This study has several strengths. We extracted data for the three most disaster-affected prefectures using a nationwide, complete, and individualized database. The proportion of people using care services, while being uncovered by LTCI is very limited, which excluded the effect of selection bias in this study. In addition, certification of disaster-affected persons was conducted by municipal governments, which produced a substantial amount of evidence supporting the physical and/or financial damage of affected persons and affected families during certification. Thus, the disaster-affected status of each individual in the dataset is reliable.

 A potential limitation of this study is the possibility that, as a reason for discontinuing LTC use, some individuals might have recovered to the point where they no longer required long-term care services. In general, however, it is very unlikely that a person requiring nursing care will become completely independent in the course of natural aging. As shown in Table 3, the proportion of those who discontinued nursing care because they became fully recovered was negligible. On the other hand, among people using in-facility services, the proportion of death cases among non-affected people was almost double that among affected people. The mechanism under which this gap takes place is unclear, and further studies are needed to reveal the real reasons for LTC discontinuation among all subjects.

Another possible limitation is the misclassification between cases and controls [38]. In this study, those who were admitted to a hospital immediately after the disaster and those who died immediately after the disaster may not have been certified as disaster-affected people, or even if they were certified as such, they may have been unrecorded in the database, which suggests the existence of a differential misclassification between disaster affected and non-affected people. However, this type of classification bias, if any, would have underestimated, not overestimated, the risk of disaster- affected persons estimated in this study, and thus would not affect the validity of our interpretation.

In addition, the higher hazard ratios for the relatively younger subgroup in the subgroup analysis may be due to the potential selection bias that younger subjects are more independent than older subjects and more likely to apply for disaster certification. However, in the 2018 torrential rain disaster, the government gave a special waiver to victims who had difficulty in applying for disaster certification, and severely affected persons did not have to pay an out-of-pocket charge for their care services if they orally reported their damage status, and the service providers recognized the status as true. Thus, we consider age was not a substantial barrier, and the degree of this selection bias would be small.

Finally, the frequency of subjects that moved from the disaster areas with and without a change in their residence registration is unknown. The former were treated as continuing and the latter as discontinuing LTC services in this study, although many of the latter actually continued using the services. The Japanese government designated the 2018 Japan Floods as an “extremely devastating disaster,” and therefore those affected were exempted from out-of-pocket expenses for LTC services as long as they retained their registration of residence in the affected areas, even if they had actually moved out of the areas. Thus, affected persons who moved from areas after the disaster tended not to change their registration of residence. However, according to the database, the number of affected persons who moved out of the three prefectures without changing their registration of residence was less than 10 out of 2,762 affected persons. In other words, most of the movers changed their residence registration. Therefore, a bias of the results caused by this registration issue would be minimal.

## Conclusions

In conclusion, a natural disaster forced severely affected victims to stop using LTC services, probably through death, hospitalization, undesired relocation, and stoppage of service delivery by providers. Japan implemented a nationwide long-term care insurance for the public in 2001, making long-term care services available to all citizens. However, this insurance program has been unable to keep pace with the rapidly increasing numbers of service users since its inception 20 years ago, and the scale and frequency of disasters as a result of climate change has escalated to such an extent that the Japanese government needs to address the chronic shortage of LTC services. In particular, the government needs to investigate the residential capacity of in-facility services in order to prevent the emergence of “care refugees” who are unable to receive necessary care services in the event of a disaster. The same recommendation could be applied to other countries experiencing a similar development in social ageing and disaster frequency. Through these improvements, countries will be able to bolster their own care service delivery systems and develop into more sophisticated welfare societies.

## Supplementary Information

**Additional file 1.**

## Data Availability

The datasets generated and analyzed during the current study are not publicly available due to legal reasons but are available from the corresponding author, DM on reasonable request.

## Abbreviations

ADL: Activities of Daily Living
CI: Confidence Interval
HR: Hazard ratio
LTC: Long-term Care
LTCI: Long-term Care Insurance
MHLW: Ministry of Health, Labour, and Welfare
