# Supplementary material for: Discontinuation of long-term care among persons affected by the 2018 Japan Floods: a longitudinal study using the Long-term Care Insurance Comprehensive Database
Source: BMC Geriatr. 2022 Mar 1;22:168. doi: 10.1186/s12877-022-02864-4 (PMC8886770; doi:10.1186/s12877-022-02864-4)
Supplement: Supplementary file 1 — Additional file 1. [file 12877_2022_2864_MOESM1_ESM.docx]

**Supplementary Table 1**

**Classification codes for LTC service types**

| Type of LTC Service | Classification Code |
| --- | --- |
| Day care | 15, 16, 66, 72, 73, 74, 78 |
| Home visit services | 11, 12, 13, 14, 62, 63, 64, 71, 76 |
| Short-stay services | 21, 22, 23, 24, 25, 26, 27, 28, 38, 39 |
| In-facility services | 32, 33, 35, 36, 37, 51, 52, 53, 54 |
| Multifunctional services | 68, 69, 75 |
| Care manager services | 31, 34 |
| LTC, long-term care |  |

| **Supplementary Table 2**  **Hazard Ratios of Disaster Affected Persons for Discontinuation of LTC with Complete Case and Multiple Imputed Analysis** | | | | | | | |
| --- | --- | --- | --- | --- | --- | --- | --- |
|  | Complete case (N=259,081) | | |  | Imputed (N=264,612) | | |
| Hazard ratio (95% CI) | Crude | Age, Sex, Adjusted | Multivariable Adjusted* | | Crude | Age, Sex, Adjusted | Multivariable Adjusted* |
| Individual-associated factors |  |  |  | |  |  |  |
| Affected | 2.15 (2.00-2.33) | 2.15 (1.99-2.32) | 2.06 (1.91-2.23) | | 2.13 (1.98-2.30) | 2.15(1.99-2.32) | 2.06 (1.91-2.23) |
| Non-affected (ref) | Ref | Ref | Ref | | Ref | Ref | Ref |
| Age, categorical |  |  |  | |  |  |  |
| <65 | 0.93 (0.83-1.04) | 0.92 (0.83-1.04) | 0.91 (0.81-1.01) | | 1.02 (0.93-1.13) | 1.02 (0.92-1.13) | 1.00 (0.91-1.11) |
| 65-69 | Ref | Ref | Ref | | Ref | Ref | Ref |
| 70-74 | 1.03 (0.95-1.11) | 1.06 (0.98-1.14) | 1.07 (0.99-1.16) | | 1.03 (0.96-1.12) | 1.06 (0.98-1.14) | 1.08 (1.00-1.16) |
| 75-79 | 1.06 (1.00-1.15) | 1.13 (1.05-1.21) | 1.17 (1.09-1.25) | | 1.07 (0.99-1.14) | 1.13 (1.05-1.21) | 1.16 (1.09-1.25) |
| 80-84 | 1.04 (0.98-1.11) | 1.14 (1.07-1.22) | 1.19 (1.11-1.35) | | 1.05 (0.98-1.12) | 1.14 (1.07-1.22) | 1.19 (1.12-1.27) |
| 85-90 | 1.09 (1.02-1.16) | 1.21 (1.14-1.30) | 1.26 (1.18-1.35) | | 1.09 (1.02-1.16) | 1.22 (1.14-1.30) | 1.27 (1.19-1.35) |
| 90-94 | 1.24 (1.16-1.33) | 1.42 (1.33-1.51) | 1.45 (1.36-1.55) | | 1.24 (1.17-1.33) | 1.41 (1.33-1.51) | 1.45 (1.36-1.55) |
| >95 | 1.54 (1.43-1.65) | 1.80 (1.68-1.93) | 1.76 (1.64-1.89) | | 1.54 (1.44-1.65) | 1.80 (1.68-1.93) | 1.76 (1.64-1.89) |
| Men | 1.36 (1.32-1.38) | 1.45 (1.42-1.48) | 1.46 (1.43-1.50) | | 1.36 (1.33-1.39) | 1.45 (1.42-1.49) | 1.46 (1.43-1.50) |
| Level of care needs |  |  |  | |  |  |  |
| Support level 1 | Ref | Ref | Ref | | Ref | Ref | Ref |
| Support level 2 | 1.06 (1.01-1.13) | 1.06 (1.00-1.12) | 1.06 (1.00-1.12) | | 1.07 (1.01-1.13) | 1.07 (1.00-1.13) | 1.00 (0.95-1.05) |
| Care needs level 1 | 1.02 (0.97-1.07) | 0.97 (0.93-1.02) | 1.00 (0.95-1.05) | | 1.02 (0.97-1.07) | 0.98 (0.93-1.03) | 1.06 (1.01-1.11) |
| Care needs level 2 | 1.12 (1.07-1.18) | 1.06 (1.01-1.11) | 1.09 (1.03-1.14) | | 1.13 (1.07-1.18) | 1.06 (1.01-1.12) | 1.09 (1.05-1.13) |
| Care needs level 3 | 1.21 (1.15-1.27) | 1.13 (1.07-1.19) | 1.18 (1.12-1.25) | | 1.22 (1.16-1.28) | 1.14 (1.08-1.20) | 1.19 (1.15-1.24) |
| Care needs level 4 | 1.41 (1.34-1.49) | 1.32 (1.25-1.39) | 1.40 (1.33-1.48) | | 1.42 (1.35-1.50) | 1.33 (1.26-1.40) | 1.41 (1.36-1.47) |
| Care needs level 5 | 1.72 (1.63-1.81) | 1.65 (1.56-1.73) | 1.76 (1.66-1.85) | | 1.73 (1.64-1.82) | 1.65 (1.57-1.74) | 1.76 (1.69-1.83) |
| Facility-associated factors |  |  |  | |  |  |  |
| Closure of facility in use | 1.56 (1.43-1.71) | 1.56 (1.43-1.71) | 1.41 (1.29-1.54) | | 1.57 (1.44-1.71) | 1.57 (1.44-1.71) | 1.42 (1.29-1.55) |
| Type of LTC services in use |  |  |  | |  |  |  |
| Home visit service | 1.08 (1.06-1.11) | 1.09 (1.06-1.11) | 1.10 (1.07-1.13) | | 1.08 (1.06-1.11) | 1.09 (1.06-1.12) | 1.10 (1.07-1.13) |
| Day care | 0.94 (0.92-0.96) | 0.93 (0.91-0.95) | 0.922 (0.91-0.95) | | 0.94 (0.92-0.96 | 0.93 (0.91-0.95) | 0.92 (0.90-0.95) |
| Short term stay | 1.09 (1.07-1.12) | 1.07 (1.04-1.09) | 1.08 (1.05-1.11) | | 1.09 (1.07-1.12) | 1.06 (1.04-1.09) | 1.08 (1.05-1.11) |
| Nursing facilities | 1.03 (1.01-1.06) | 1.01 (0.99-1.03) | 0..86 (0.83-0.88) | | 1.03 (1.01-1.06) | 1.01 (0.99-1.03) | 0.85 (0.83-0.88) |
| Multifunctional facility | 1.02 (0.96-1.08) | 1.02 (0.97-1.09) | 1.04 (0.98-1.11) | | 1.02 (0.96-1.08) | 1.03 (0.97-1.09) | 1.05 (0.99-1.11) |
| Care manager service | 1.05 (1.01-1.08) | 1.03 (1.00-1.06) | 0.98 (0.95-1.02) | | 1.05 (1.01-1.08) | 1.03 (1.00-1.06) | 0.98 (0.95-1.01) |
| Region-associated factors |  |  |  | |  |  |  |
| Population density per 10m^2^ | 1.00 (0.99-1.00) | 1.00 (0.99-1.00) | 1.00 (1.00-1.02)- | | 1.00 (0.99-1.00) | 1.00 (0.99-1.00) | 1.00 (0.99-1.00) |
| Hospital beds per 100 population | 1.00 (0.98-1.01) | 1.00 (0.98-1.01) | 1.00 (0.98-1.01) | | 1.00 (0.98-1.02) | 1.00 (0.98-1.01) | 1.00 (0.99-1.00) |
| No. of nursing facilities per 1000 older people | 0.87 (0.32-2.38) | 0.46 (0.17-1.26) | 0.87 (0.77-0.99) | | 0.99 (0.89-1.09) | 0.93 (0.84-1.03) | 0.88 (0.78-1.00) |
| Average income (every 1million yen) | 0.99 (0.89-1.09) | 0.93 (0.84-1.02) | 1.10 (1.02-1.19) | | 0.97 (0.93-1.01) | 1.00 (0.96-1.04) | 1.10 (1.02-1.19) |
| Proportion of the elderly (%) |  |  |  | |  |  |  |
| Low 19.8-25.0 | Ref | Ref | Ref | | Ref | Ref | Ref |
| Middle 25.5-30.7 | 1.09 (1.06-1.12) | 1.08 (1.06-1.11) | 1.12 (1.09-1.16) | | 1.09 (1.06-1.12) | 1.08 (1.05-1.11) | 1.12 (1.09-1.16) |
| High 31.2-49.1 | 1.07 (1.04-1.10) | 1.05 (1.02-1.08) | 1.14 (1.09-1.19) | | 1.07 (1.04-1.10) | 1.05 (1.02-1.08) | 1.14 (1.09-1.19) |
| LTC, long-term care; CI, confidence interval; ref, reference; *Adjusted for age, sex, level of care needs, LTC service in use, facility closure, population density, average income, elderly proportion | | | | | | | |
